# The Kelling Sanatorium, Holt, Norfolk

**Published:** 1903-11-07

**Authors:** 


### THE KELLING SANATORIUM, HOLT, NORFOLK.

HOLT is a station on the Great Northern line, and is about four-and-a-half hours' run from King's Cross. The sanatorium is a mile-and-a-half or so from the Holt Station. The main part of the sanatorium was formerly an old farmhouse, and still retains much of the charm of bygone days, having nice old-fashioned lawns and flower-beds with their trim edgings of dwarf box. Many alterations have been made to make it suitable for its present purpose, and among others the old barn has been turned into a dining hall large enough to seat 35 patients, and cubicles have been erected entirely separate but close to the old building. At present, with rooms and cubicles, it can accommodate about twenty patients, and foundations for other cubicles have been got out. These new ones will give a total of 11 additional beds. As funds permit it is intended to erect more of these, and then the old building will cease to be used for patients, and will be devoted entirely to the staff so far as sleeping accommodation is concerned. We believe that this system foreshadowed, that is sleeping in cubicles, or huts, or kiosks, is the correct principle on which to found the modern treatment of tuberculosis, which treatment, as Dr. Fanning, the Resident Medical Officer, points out, should aim at as near an approach as possible to sleeping in the open air, and he states that he has noticed cases where the fever did not abate when the night was spent in ordinary sanatorium rooms with open windows (although the whole day was passed out of doors), but which yielded when the night was spent in cubicles. This is a statement which can be easily accepted, and it makes us doubt the wisdom of building large and expensive sanatoria containing rows of bedrooms. Undoubtedly single-bedded châlets, exposed on all sides to the air, would be the ideal provision to make were it not that this system would involve considerable inconvenience, and therefore increased cost of maintenance. It is further pointed out that the ordinary hospital ward can give no adequate supply of air to the phthisical patient. The cubicles must be arranged so as to ensure the maximum of air, with facility of service, economy of maintenance, and, what is often of much importance, economy of construction.

For such sanatoria as he manages Dr. Fanning advises the erection of châlets, which can be built for about £60 for four beds. This plan is described in a late number of "Tuberculosis," from which we have extracted some of the above particulars, and as we have also inspected the cubicles at the Kelling Sanatorium we are able to state that they have every appearance of efficiency and suitableness, and that they may be confidently recommended wherever economy of construction is of importance, and it generally is of importance when the sanatorium is intended for the lower fringe of the middle class.

The patients at the Kelling Sanatorium pay 30s. a week when this sum can be reasonably afforded; if not donations are given from the charitable fund in the hands of the managing committee, so that this institution is intended to help those who cannot pay the higher fees which must be charged at purely private sanatoria. This is exactly what is so urgently wanted at the present moment. When it is realised that the number of beds at disposal in this and in similarly constituted sanatoria is hopelessly short of the demand on it, and when it is known, as Dr. Fanning shows, that something like £100 would build and equip a cubicle for four patients, it can hardly be doubted that charitably disposed individuals will come forward and provide what is a most pressing want. One generous man presented the original building to the Kelling managers, others have endowed beds, but the institution is still without a laundry or a dairy, and we venture to hope that special donations will soon be forthcoming to build these essential adjuncts.

The modern treatment is so much identified with perfect rest that it can hardly fail to have a tendency to induce laziness in those cases where cure has been effected, and this is to be regretted in the majority of cases treated in sanatoria for this class of patients, as on recovery and discharge they are practically compelled to resume their former occupations. To combat this tendency Dr. Fanning encourages some light occupation after treatment has been successfully pursued for a few weeks, and he assures us that he has never seen harm from this, but often much benefit. To us this seems a much more enlightened view to take than that promulgated by some physicians who would deprecate any employment whatever.

